# In Vitro Models of Cell Senescence: A Systematic Review on Musculoskeletal Tissues and Cells

**DOI:** 10.3390/ijms242115617

**Published:** 2023-10-26

**Authors:** Francesca Veronesi, Deyanira Contartese, Laura Di Sarno, Veronica Borsari, Milena Fini, Gianluca Giavaresi

**Affiliations:** 1 Surgical Sciences and Technologies, IRCCS Istituto Ortopedico Rizzoli, Via di Barbiano 1/10, 40136 Bologna, Italy; francesca.veronesi@ior.it (F.V.); laura.disarno@ior.it (L.D.S.); veronica.borsari@ior.it (V.B.); gianluca.giavaresi@ior.it (G.G.); 2 Scientific Direction, IRCCS Istituto Ortopedico Rizzoli, Via di Barbiano 1/10, 40136 Bologna, Italy; milena.fini@ior.it

**Keywords:** ageing, cell senescence, musculoskeletal system, in vitro models, systematic review

## Abstract

Ageing is an irreversible and inevitable biological process and a significant risk factor for the development of various diseases, also affecting the musculoskeletal system, resulting from the accumulation of cell senescence. The aim of this systematic review was to collect the in vitro studies conducted over the past decade in which cell senescence was induced through various methods, with the purpose of evaluating the molecular and cellular mechanisms underlying senescence and to identify treatments capable of delaying senescence. Through three electronic databases, 22 in vitro studies were identified and included in this systematic review. Disc, cartilage, or muscle cells or tissues and mesenchymal stem cells were employed to set-up in vitro models of senescence. The most common technique used to induce cell senescence was the addition to the culture medium of tumor necrosis factor (TNF)α and/or interleukin (IL)1β, followed by irradiation, compression, hydrogen peroxide (H_2_O_2_), microgravity, in vitro expansion up to passage 10, and cells harvested from damaged areas of explants. Few studies evaluated possible treatments to anti-senescence effects. The included studies used in vitro models of senescence in musculoskeletal tissues, providing powerful tools to evaluate age-related changes and pathologies, also contributing to the development of new therapeutic approaches.

## 1. Introduction

Human ageing is an irreversible and inevitable biological process characterized by the physiological deterioration of physical functions and an increasing of pathological conditions starting from the third decade of age [1,2]. In the Western population, the number of people over 60 years old is increasing, and it is forecasted to triple by 2050 [3].

Ageing is a significant risk factor for the development of various diseases, also affecting the musculoskeletal system, with factors such as intervertebral disc degeneration (IDD), osteoarthritis (OA), osteoporosis (OP), and sarcopenia. These pathologies result from the accumulation of cell senescence and degenerative stimuli, both from inside the cell and from the microenvironment, contributing to progressive tissue degeneration [4]. Cell senescence is defined as a permanent state of cell cycle arrest in which cells become resistant to mitogenic stimuli. Senescence can act as a physiological process occurring throughout the lifespan, which prevents the replication of cells with damaged DNA, playing an important role during development and wound healing and providing an antitumorigenic function [4].

During ageing, an imbalance arises between the production of reactive oxygen species (ROS) and the ability to repair tissue damage through endogenous antioxidant defenses [5], causing an increase in the oxidative stress. This oxidative stress, in turn, adversely affects cell function (causing damage to proteins, nucleic acids, and lipids), reduces the tissue repair capability, and induces cellular senescence. Additionally, senescent cells prompt neighboring cells to undergo senescence through the paracrine signaling pathway [6].

Two modalities of induction of cellular senescence are known: the replicative senescence and the stress-induced premature senescence (SIPS). Cell senescence is caused, in the first case, by telomere attrition after repeated proliferation while, in the second case, by subtoxic doses of extrinsic or intrinsic stimuli such as irradiation, oxidative stress, drugs, oncogenic stress, and metabolic or epigenetic changes [7].

When a cell becomes senescent, its proliferation and replication cease [8], leading to a stable cell cycle arrest [7,9]. Senescent cells start producing pro-inflammatory and matrix-degrading molecules, including metalloproteinases (MMPs) and Tumor Necrosis Factor α (TNFα), collectively known as the Senescence-Associated Secretory Phenotype (SASP) [10,11]. Moreover, senescent cells are characterized by morphological changes, chromatin re-organization, mitochondrial dysfunctions, and altered gene expression. 

Recent hypotheses suggest that eliminating senescent cells, possibly using chemical agents like senolytic compounds, can improve tissue homeostasis and delay age-associated pathologies [12].

Over the past decade, in vitro models that simulate specific pathologies or scenarios have gained prominence. These models align with the 3R (reduction, refinement, and replacement) principles for animal use for scientific purposes because they are performed using advanced in vitro systems rather than in vivo ones [13]. Actually, several in vitro culture systems reproduce the characteristics of many pathologies, including complex conditions like cancer [14].

In the literature, some in vitro models are usually employed to induce cell or tissue senescence: (1) primary cells cultured in monolayers under senescence-inducing conditions; (2) three-dimensional (3D) tissue models (such as organoids, spheroids, and tissue-engineered constructs), which better replicate the native tissue microenvironment compared to cell cultures [15]; (3) cultures in bioreactors simulating dynamic conditions such as mechanical loading or fluid shear [16]; (4) co-culture models involving multiple cell types to study their mutual influence [17]; and (5) genetically modified cells or tissues [18].

The aim of this systematic review was to collect the in vitro studies conducted over the past decade in which cell senescence was induced through various methods. The objective was to evaluate the molecular and cellular mechanisms underlying senescence and to identify treatments capable of delaying senescence.

This review investigates different techniques to induce senescence in musculoskeletal cells and tissue cultures, as well as the in vitro methodologies used to assess cell senescence. The perspective of our review is to encourage further research exploring the characteristics of senescent cells and potential therapeutic treatments in this context.

## 2. Materials and Methods

### 2.1. Eligibility Criteria

For the selection and analysis of the relevant papers, a PICO question (Population of interest (P), Intervention (I), Comparators, and Outcomes (CO)) was formulated.

The Population considered was in vitro studies in which musculoskeletal system cells or tissues were employed. The Intervention was all in vitro cultures in which cell or tissue senescence was induced. The Comparator was cells cultured in normal conditions, without the induction of senescence. The considered primary Outcome was cell senescence evaluation through cellular, molecular, biochemical, and histological techniques. In addition, a secondary outcome was the effect of some treatments used to reduce cell senescence when present.

### 2.2. Search Strategy

The search was performed on 15 July 2023 and included research published from 15 July 2013 to 15 July 2023 according to the Preferred Reporting Items for Systematic Reviews and Meta-Analyses (PRISMA) statement.

Three electronic databases (PubMed, Scopus and Web of Science) were used to identify relevant papers using the following MESH with Boolean operators: ((“In Vitro Models”[Mesh]) AND (“Aging”[Mesh] OR “Cellular Senescence”[Mesh])) AND (“Musculoskeletal System”[Mesh]). The limit was the language (English) for all the databases.

After the removal of duplicates (Mendeley 1.14, www.mendeley.com, accessed on 17 July 2023), relevant articles were screened using the title and abstract by two reviewers (FV and DC), and articles that did not meet the inclusion criteria, previously reported, were excluded. The included full-text articles were retrieved and reviewed by the two authors, and any disagreement was resolved through discussion until consensus was reached or with the involvement of a third author (VB). The researchers involved in the process of reviewing the papers used an Excel spreadsheet to independently perform the screening and data extraction. The following information was extracted from each paper to summarize the evidence reported in each study: (a) cells/tissues, (b) in vitro model of senescence, (c) evaluations, (d) main results, and (e) references (Ref.) (Table 1).

## 3. Results

**Figure 1:**
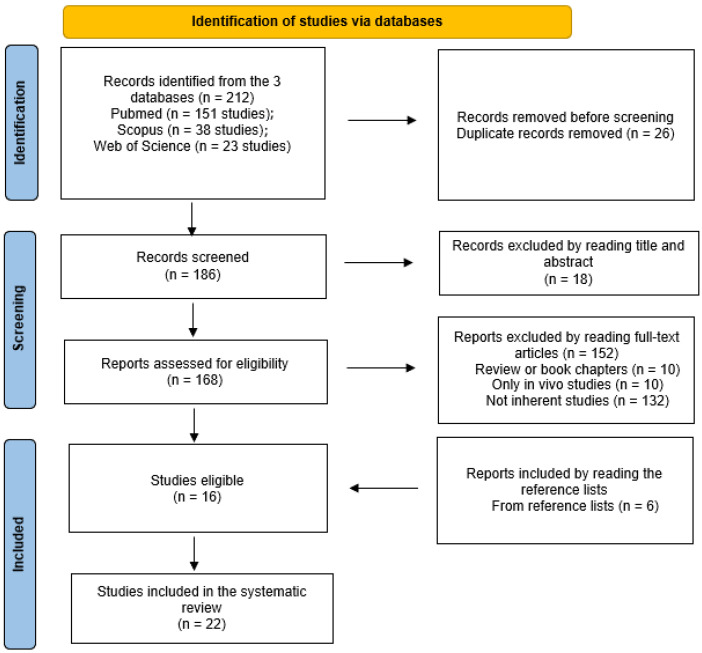
Flow-chart of the included studies according to PRISMA principles.

The initial literature search retrieved 151 studies from PubMed, 38 from Scopus, and 23 from Web of Science, for a total of 212 articles. There were 186 identified papers after the removal of duplicates (26 records). Reading the titles and abstracts, 18 papers were excluded, and 168 full texts were evaluated for inclusion. Reviews or book chapters (n = 10), in vivo studies (n = 10), and non-inherent studies because they employed cells from other tissues different from musculoskeletal ones, or because the authors did not induce cell senescence (n = 132), were excluded, for a total of 152 studies. The remaining 16 articles were considered eligible. A further search was performed by reading the reference lists of these eligible articles, and a further six papers were added. Therefore, a total of 22 articles were included in the present systematic review (Figure 1).

In the studies included in this review, nucleus pulposus (NP), annulus fibrosus (AF) cells or discs [19,20,21,22,23,24,25,26,27,28,29,30], chondrocytes [31,32,33,34,35,36], myoblasts [2,37,38], and mesenchymal stem cells from bone marrow (BMSCs) [36] or from NP (NPMSCs) [39] were employed to set-up in vitro models of senescence.

### 3.1. In Vitro Techniques to Evaluate Cell Senescence

In vitro cell senescence was evaluated through the assessment of cell proliferation, apoptosis, and cell cycle phases [2,20,23,24,25,27,28,29,30,31,34,35,36,37,38]; beta Galactosidase (SA-β-Gal) staining [20,21,22,23,24,25,27,28,29,30,31,32,33,34,36,37,38,39]; protein production [19,20,21,22,23,24,25,26,27,28,29,31,32,33,34,35,36,37,38,39]; gene expression [21,22,23,24,25,27,28,29,30,31,32,33,34,35,36,38]; telomere length and telomerase (TE) activity [25,27,29,30,33,34,35,36]; glycosaminoglycan (GAG) and hydroxyproline (HYP) content [25,29,30,31,32,33]; and ROS production [26,27,29,34,36].

### 3.2. Nucleous Pulposus/Annulus Fibrous Cells and Disc Tissue

Most studies (12/22 studies) employed NP or AF cells or discs harvested from rats, pigs, humans, bovines, and rabbits [19,20,21,22,23,24,25,26,27,28,29,30].

#### 3.2.1. Pro-Inflammatory Cytokines

In four studies, cells or discs were treated with pro-inflammatory cytokines, such as TNFα and/or interleukin1β (IL1β) [19,20,21,22].

More precisely, the addition of both TNFα (20 ng/mL) and IL1β (20 ng/mL) to the culture medium increased the ROS, SA-β-Gal, p16, p53, and degrading enzymes’ gene expressions and reduced cell proliferation, TE activity, and matrix proteins. In addition, this combination also altered the distribution of cells across cell cycle phases; the percentage of cells in G0/G1 phase was higher than that in S phase [19].

Another study focused solely on TNFα, using concentrations of 10 ng/mL for cells and 200 ng/mL for disc cultures. The authors observed reduced cell viability, TE activity, and matrix gene expressions and increased SA-β-Gal activities, ROS levels, and p53 and p16 gene expressions [20].

A study tested different TNFα concentrations (10, 20, and 40 ng/mL) in cell culture, observing dose-dependent decreases in cell proliferation, TE activities, and matrix proteins, as well as dose-dependent increases in p53, p16, and AKT gene expressions. Similar results were observed in groups treated with TNFα for 3 days with 3 days of recovery or with six consecutive days of treatment [21].

Finally, a 3D co-culture model was set-up with NP cells, cultured in presence of TNFα (20 ng/mL), and calcium alginate gel balls, empty or containing normal BMSCs or NP cells.

The addition of TNFα increased SA-β-Gal activities in NP cells, whereas the addition of balls with BMSCs reversed the senescence phenotype. This intervention reduced MMP9 protein expression and p16, p21, and p53 gene expressions and increased zinc metallopeptidase STE24 (ZMPSTE24) protein levels [22].

#### 3.2.2. Mechanical Stimuli

Four studies induced senescence in discs, NP, and AF cells through intermittent, dynamic, or static compression and elongation [23,24,25,26].

Discs were loaded in bioreactors with intermittent compression at two different intensities (0.1 and 1.3 MP), showing higher SA-β-gal activities, elevated expressions of p16, p21, p53, catabolic enzymes, and p38 proteins, and reduced matrix proteins and tissue inhibitors of metalloproteinase TIMP3 gene expression at 1.3 MP than at 0.1 MP [23].

In addition, cells that underwent 20% dynamic compression (1.0 Hz for 6 h/day) had reduced proliferation, increased SA-β-Gal activity, and expressions of p53, p16, nuclear factor kappa-light-chain-enhancers of activated B cells (NF-κB), and ROS proteins [24]. 

One study compared static and dynamic compression (0.4 MP, 1.0 Hz for 4 h/day for 7 days) in disc culture, observing that static compression led to greater SA-β-gal, p16, and p53 protein expressions and reduced TE activity and matrix proteins compared to dynamic compression. Notably, dynamic compression seemed to revert the senescent phenotype [25].

Moreover, two different magnitudes (5% and 20% elongation) of mechanical tension (1 Hz for 8 h/day for 12 days) were applied to AF cells. Cell proliferation, TE activity, S phase, matrix, and BCL1 and LC3 proteins were significantly reduced, whereas G0/G1 phase and p16 and p53 proteins were significantly increased, with 20% more elongation than with the 5% one [26]. 

#### 3.2.3. Hydrogen Peroxide

In two studies, hydrogen peroxide (H_2_O_2_) added to NP cells at different concentrations (from 0.05 to 2 mM) increased ROS levels, SA-β-Gal activities, p53, p21, and p16 protein expressions, and pro-inflammatory cytokines’ gene expressions, accompanied by reduced cell viability and proliferation in a dose-dependent manner [27,28], with significantly detrimental effects at concentration of 0.5 mM [28].

#### 3.2.4. Irradiation

Finally, two studies irradiated human and rat NP or AF cells with γ-rays (^60^Co gamma at a rate of 2 Gy/min or 2.5 Gy/min) [29,30]. In the first study, irradiation reduced cell proliferation and increased SA-β-Gal and GL13 protein levels. This effect was more conspicuous in cells harvested from old rats or patients compared to those from young rats or healthy subjects [29].

In the second study, irradiations were given to cells cultured in two different media: the first composed by 10% fetal bovine serum (FBS), 4.5 mg/mL glucose, 300 mOsm/Kg H_2_O, and 20% O_2_, whereas the second consisted of serum-free 0.9 mg/mL glucose, 400 mOsm/Kg H_2_O, and 2% O_2_. Both media increased p16, p21, and MMP gene expressions and reduced collagen II (COLL II) gene expression. Additionally, the first medium also increased SA-β-Gal activity and reduced cell proliferation [30].

### 3.3. Chondrocytes

The second larger group of studies (6/22 studies) employed chondrocytes obtained from humans [31,32,34,36], horses [32], rats [33,35], and rabbits [35].

#### 3.3.1. Irradiation

Three of these studies evaluated the effects of irradiations on chondrocyte senescence [31,32,33].

The results revealed that γ-rays (at 10 Gy) induced SA-β-gal, p16, and yH2AX proteins and that the addition of transforming growth factor beta 1 (TGFβ1) and basic fibroblastic growth factor (bFGF) further increased SA-β-gal, p16, yH2AX proteins, insulin-like growth factor binding protein 3 (IGFBP3), and C-C motif chemokine ligand 2/monocyte chemoattractant protein 1 (CCL2) gene expressions [32]. Similarly, γ-rays (at 1.080 Gy) stimulated high SA-β-gal, IL6, and MMP13 gene expressions and reduced proliferation, Caspase-3 protein, Bax/Bcl2, and COLL II gene expressions in chondrocytes. When these irradiated chondrocytes were co-cultured with BMSCs, they reduced proliferation, COLL II, and aggrecan (AGG) proteins and increased Caspase-3 protein, Bax/Bcl2, octamer-binding transcription factor 4 (OCT4), and NANOG gene expressions and SA-β-Gal activities in BMSCs [33].

Furthermore, a study compared the effects of monoenergetic C-ions (75–95 MeV/mm) with X-rays (225 kV) on chondrocytes cultured in a monolayer or in a collagen scaffold [31]. The authors observed that C-ions significantly reduced chondrocyte proliferation more than X-rays in monolayer cultures. In both monolayer and in collagen scaffold cultures, a higher increase in SA-β-gal expression was observed with C-ions than with X-rays [31].

#### 3.3.2. Pro-Inflammatory Cytokines

Two studies treated chondrocytes with IL1β (10 ng/mL) [34,35]. The first study confirmed that IL1β reduced gene expression of COLL II while increasing ADAMTS5 gene expression, as well as MMP13 and 78-KDa glucose-regulated protein (GRP78) levels [34]. In the second study, the combination of lithium chloride (LiCl) (20 mM) and IL1β led to greater increases in β-catenin, MMP13, p53, and sirtuin-1 (SIRT1) proteins compared to the combination of dickkopf-1 (DKK1) (100 ng/mL) and IL1β. In addition, Wnt-1 (10 ng/mL) increased cell senescence and p53 and p16 proteins, and, when paired with IL1β, it increased MMP3 and MMP13 proteins [35].

#### 3.3.3. Isolation from Damaged Cartilage

Finally, micromasses of chondrocytes harvested from severely damaged cartilage areas displayed lower cartilage matrix depositions, telomere lengths, matrix gene expressions, and higher MMP13, p16, p21, and p53 protein levels than chondrocytes isolated from preserved cartilage areas [36].

### 3.4. Myoblasts

Other studies (3/22 studies) used C2C12 cell lines [2,37] or purchased human myoblasts [38] for their investigations.

#### 3.4.1. Serum from Elderly Patients

Myotubes derived from C2C12 cells were treated with 10% serum from older donors, showing lower myotube diameters and decreased nuclear factor I (NFI), AKT, p70S6K1 and eukaryotic translation elongation factor 2 (eEF2) proteins than myotubes cultured in sera from younger subjects [37]. Similar results were obtained when the same cells were cultured in the presence of 5% plasma from older subjects, showing higher scratch sizes and lower cell diameters and proliferation rates than cells cultured with 5% plasma from younger subjects [2].

#### 3.4.2. Microgravity

Finally, human myoblasts at the first passage were cultured under normal gravity (1 G), microgravity (1 × 10^−3^ G; µG), or normal gravity at the fifth passage. Microgravity decreased myosin heavy chain (MHC) and desmin proteins, cell proliferations, and myotubes’ diameters, whereas it increased the myogenic factor 5 (Myf5) protein. Both microgravity exposure and the fifth in vitro passage led to increased SA-β-Gal activity [38].

### 3.5. Mesenchymal Stem Cells 

MSCs were employed in 2/22 studies [36,39]. Both BMSCs and NPMSCs were obtained from patients.

#### 3.5.1. Long Culture Passages

BMSCs were maintained in culture until the 10th passage, displaying lower proliferation rates, differentiation potentials, telomere lengths, chondrogenic differentiations, and higher p16, SA-β-gal, MMP13, p21, and p53 protein levels than BMSCs cultured for four in vitro passages [36].

#### 3.5.2. Pro-Inflammatory Cytokines

TNFα (10 ng/mL) added to NPMSCs cultures increased senescent cell morphologies, SA-β-Gal activities, and p16, p21, p53, IL6, MMP13, and ADAMTS5 gene expressions [39].

### 3.6. Senescence Treatments

As shown in Table 2, 5/22 studies also evaluated potential treatments aimed at reducing cell senescence in the above-mentioned in vitro models [19,20,24,34,39]. Among these studies, three focused on NP cells [19,20,24], one on NPMSCs [39], and one on chondrocytes [34].

In the context of senescent NP cells, treatment with resveratrol (at concentrations of 30, 60, or 100 µM) was administered. This intervention resulted in reductions in ROS, SA-β-gal activities, expressions of p16, p53, catabolic enzymes, and NF-κB proteins, as well as a decrease in cells present in the G0/G1 phase. Additionally, resveratrol increased cell proliferation, TE activity, matrix proteins, and cells in the S phase [19,24].

In another study, treating senescent NP cells with 17-beta-estradiol (E2) (10^−7^ M) enhanced cell proliferation, TE activity, matrix protein levels, and reduced SA-β-Gal activity, ROS levels, as well as p53 and p16 protein expressions [20].

The addition, co-cultures of notochordal cell (NC)-rich NP explants increased proliferations, expressions of carbonic anhydrase 12 (CA12), FOXF1, Pax-1, and matrix proteins, and decreased COLL I protein levels of senescent NPMSCs [39].

Finally, senescent chondrocytes were cultured in the presence of 100 mM 4-phenylbutyric acid (4-PBA). The authors observed a decrease in GRP78 protein levels, SA-β-gal activity, cell apoptosis, and an increase in cell viability after treatment [34].

## 4. Discussion

This systematic review collected and analyzed in vitro studies from the literature that evaluated the existing in vitro models used to induce senescence in musculoskeletal cells and tissues over the last 10 years.

Generally, an in vitro environment can provide information about cell–cell and cell–extracellular matrix interactions, expressions of surface receptors, and syntheses of proteins and growth factors. By increasing the in vitro model’s complexity, we are able to represent and recapitulate the characteristics of many pathologies through the use of cells or tissues from affected subjects. Moreover, complex in vitro models are functional for the gathering of preliminary data on the safety and cytocompatibility but also on the bioactivity and therapeutic efficacy of newly developed materials, medical devices, or therapies [40].

**Figure 2:**
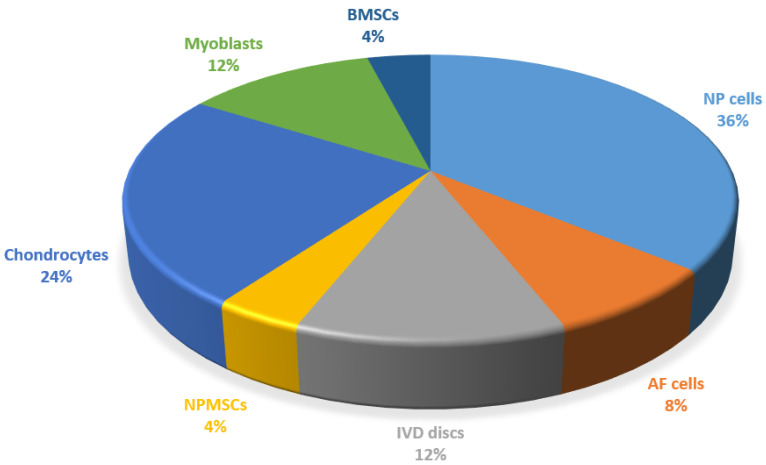
Percentage of each cell and tissue type employed in the studies of the review.

In the present systematic review, 22 studies were found, and most of them regarded cells or tissues obtained from intervertebral disc (IVD) [19,20,21,22,23,24,25,26,27,28,29,30], followed by chondrocytes [31,32,33,34,35,36], myoblasts [2,37,38], and MSCs [36,39] (Figure 2).

Almost all the cells and tissues utilized in these studies were harvested from healthy animals (15/22) or human subjects (6/22). Rats were the most employed donors of cells [19,20,21,22,24,26,27,29,33,35], followed by pigs for disc cultures [23,25], bovines [30], horses [32], and rabbits [35]. One important thing to mention is that the ages of these animals were quite young: rats (from 4–5 days to 3 months); pigs (from 3 months to 13 months); bovine (age not reported); horses (4–7 years); and rabbits (1 month).

Primary human cells were collected from IVD or cartilage of healthy subjects [28,29,34], from cartilage of patients affected by OA [36] or cadavers [32], and from patients with lumbar disc herniation [39]. The range of ages of these subjects was from 28 to 73 years, even if some studies did not indicate the age.

At last, purchased cell lines of humans and mice were also employed, and they were chondrocytes [31], C2C12 cells [2,37], and myoblasts [38].

In the studies of the present review, the above-mentioned cells and tissues were cultured prevalently in two-dimensional (2D) monolayers [2,19,20,21,22,26,27,28,29,30,31,32,33,34,35,36,37,38,39]. Other types of in vitro models concerned the culturing of IVD discs [20,23,25], NP cells suspended in collagen solution and seeded into decalcified bone matrixes (DBMs) [24], and chondrocytes cultured in collagen scaffolds [31] or as micromasses [36].

**Figure 3:**
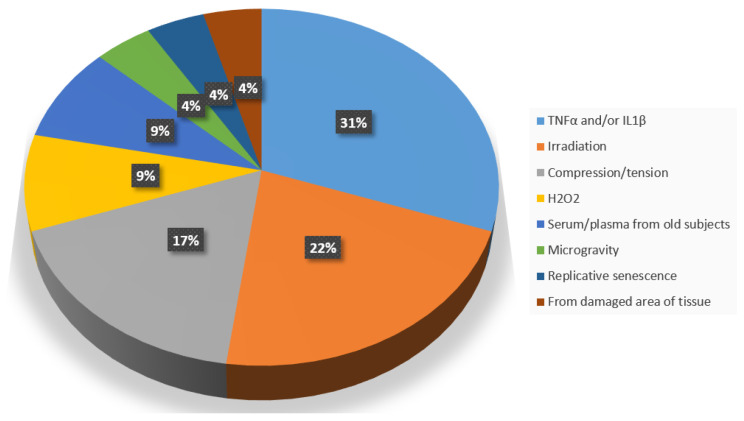
Percentages of the different methods to induce cell or tissue senescence in vitro.

These cells and tissues were induced to senescence by various methods (Figure 3), each of which had pros and cons, the most common of which was the addition to the culture medium of TNFα (10–40 ng/mL, 200 ng/mL) and/or IL1β (10, 20 ng/mL) [19,20,21,22,34,35,39] in NP cells and discs [19,20,21,22], chondrocytes [34,35], and NPMSCs [39].

TNFα and IL1β are the two inflammatory cytokines often used to recreate an inflammatory microenvironment in some in vitro models of tissue degeneration, including OA and disc degeneration [41,42,43]. In addition, according to the literature, the two cytokines induce cell senescence in some cell types, concluding that inflammation response-mediated cell senescence plays an important role in accelerating tissue degeneration [44] and can provoke an oxidative stress that drives cells to premature senescence. However, as the inflammatory factors were maintained throughout the experiment, the inflammation environment may exhibit a direct effect but not a long-term effect. In addition, the concentration of inflammatory cytokines used in in vitro senescence models is remarkably higher than that in human knee joints.

Secondly, NP or AF cells [29,30] and chondrocytes [31,32,33] underwent irradiation with γ-rays or monoenergetic C-ions to induce senescence.

Their use to induce senescence in cells is based on ionizing radiation, such as γ-irradiation, which is a principal DNA-damaging agent [30], and C-ion irradiation, which induces healthy organ deterioration [45]. On the other hand, the biologic effects measured in 2D cultures might be overexaggerated compared with the reality of clinics.

Then, intermittent, dynamic, or static compressions of discs and NP cells [23,24,25] and elongations of AF cells [26] were also employed. Indeed, it was observed that the various magnitudes of mechanical load, to which IVD was subjected every day during the daily activities, affected disc cell viability and biological function [46]. The non-physiological loads could accelerate disc degeneration [47], and the excessive and continuous mechanical compression may have aggravated the cellular senescence of the discs. Only one study that evaluated elongation in the induction of cell senescence used AF cells [26]. Tears and fissures of AF tissues were related with aggravations of disc degeneration [48], and the mechanical tension was considered a reason for AF structural destruction. However, the signaling pathways involved in the mechanical induction of senescence are not yet well known, and this technique is limited to a specific setting through the use of the appropriate bioreactor and appropriate stimuli.

Other authors cultured NP cells with H_2_O_2_ at different concentrations [27,28] and myotubes or myoblasts with sera or plasma obtained from old individuals [2,37].

Indeed, H_2_O_2_ is a commonly used inducer of stress-induced premature senescence and DNA damage, given its role as a natural inducer of oxidative stress [49]. However, H_2_O_2_ treatment creates a short-period senescent phenotype, which leads to rapid apoptosis and cell death in days and easily triggers cell apoptosis rather than cell senescence. 

The ageing process is associated with changes in circulating endocrine factors, such as growth factors hormones and pro-inflammatory cytokines, which are known to moderate muscle mass or the Klotho factor [50]. Unfortunately, this type of model cannot be standardized, as it incurs the bias that the sera of the donors have different amounts of the above-mentioned circulating factors.

One study employed microgravity on myoblasts [38] because microgravity causes a remarkable reduction in muscle mass and motor skills of astronauts [51]. However, this type of model is difficult to be replicated, thus providing controversial results. Furthermore, it seems that it is more a model of disuse than of senescence. 

A study proposed to create a senescence phenotype in BMSCs by an extensive in vitro expansion up to passage 10 [36], the so called “replicative senescence” [36], and another one using cultures of chondrocytes isolated from damaged areas of osteoarthritic cartilage explants, as OA shares some common mechanisms to senescence, such as low-grade inflammation and telomere shortening [36]. In contrast to the previous in vitro models that resulted in rapid apoptosis and cell death, the model of “replicative senescence” induced cell senescence without the addition of external agents. Also, this model showed a limit because the excessive cell division may not represent the actual reason causing cell senescence, and the “replicative senescence” is just one type of reasons leading to senescence.

To summarize, the above-mentioned in vitro models of senescence can be essentially divided into two groups: stress-induced premature senescence and replicative senescence. Irradiation, mechanical stimuli, the addition of H_2_O_2_ or sera, and microgravity are part of the first group, whereas the addition of inflammatory cytokines and several in vitro passages expansion belong to the second group. 

Several different techniques, both cellular and molecular ones, were employed to evaluate cell senescence. The technique of choice for the evaluation of senescence in vitro remains the assessment of cell proliferation, viability, and apoptosis [2,19,20,21,22,24,26,27,28,29,30,31,33,34,36,38,39]. Secondly, several authors evaluated the production of Sa-β-Gal using a commercial staining kit, immunostaining, and Western blot [19,20,21,22,23,24,25,27,29,30,31,32,33,34,35,36,38,39]. It is considered a key biomarker of cellular senescence because it is a hydrolase enzyme that catalyzes the hydrolysis of β-galactosides into monosaccharides only in senescent cells and distinguishes senescent cells based on the high lysosomal activity [52]. 

Then, the production (through enzyme-linked immunosorbent assay, Western blotting, immunohistochemistry, immunofluorescence and immunocytochemistry) [19,20,21,22,23,24,25,26,27,28,29,31,32,33,34,35,36,37,38,39] and expression (through real time PCR) [19,20,21,22,23,24,25,26,27,28,30,32,33,34,35,36,39] of genes encoding for matrix proteins, pro-inflammatory cytokines, and degrading enzymes were performed. Among them, particular attention should be paid to the senescence markers p53 and p16. The p53–p21 pathway represents replicative senescence, whereas the p16 pathway is linked to stress-induced premature senescence, the two mechanisms responsible for the induction of cellular senescence [53]. The p53 is a transcription factor that plays a critical role in cellular responses to stress; it is activated by DNA damage, leading to cell growth arrest, cellular senescence, or apoptosis, thereby maintaining genome integrity [54]. The p16 tumor suppressor and cell cycle regulator are necessary for the cells to keep the state of proliferative arrest over time. The expression of p16 is increased in senescent cells, as well as during natural aging or age-related pathologies [55]. 

Last but not least, some studies also evaluated TE activity [19,20,21,24,25,26] and ROS content [19,20,24,27,28].

TE is a protein that adds a telomere repeat sequence to the 3′ end of telomeres, regions of repetitive sequences at each end of the chromosomes that protect the end of the chromosome from DNA damage. During aging, TE activity is reduced, causing a shortening of telomere [56]. Its activity was evaluated through enzyme-linked immunosorbent assay (ELISA) in these studies. In regard to ROS, they induce oxidative damage to proteins, DNA, and lipids, promoting cell senescence, tissue destruction, and inflammation. They directly affect the rate of telomere shortening, further accelerating cell senescence [57].

As the phenotype of senescent cells displays variability, a single unambiguous biomarker for the identification of senescent cells has not been recognized yet. A combination of multiple biomarkers is usually accepted to distinguish between senescent and quiescent or terminally differentiated cells, reflecting the dynamicity of the senescence process. In addition, some biomarkers are not specific to senescent cells and can be present also in apoptotic cells or quiescent cells, for example. This represents a limitation in the isolation and characterization of senescent cells and a challenge for future research.

The purpose of performing in vitro studies with senescence-induced cells is to find and test agents with the ability to counteract senescence.

In the present review, just a few studies (5/22 studies) evaluated possible treatments to that effect: resveratrol [19,24] and E2 [20] in NP cells, NC-rich NP explant in NPMSCs [39], and 4-PBA in chondrocytes [34].

Resveratrol is a natural phenol produced by several plants in response to injury, such as attack of bacteria or fungi, and shows protective effects, including anti-inflammatory and anti-aging properties [58]. In the analyzed studies, resveratrol reduced ROS production, SA-β-Gal activity, p16 and p53 proteins’ production, and increased cell proliferation in senescent NP cells [19,24].

As NP cells contain estradiol receptor (ER) [59], it was observed that cell proliferation and TE activity increased, whereas SA-β-Gal activity, ROS levels, and p53 and p16 proteins decreased after exposure to E2 [20].

Finally, 4-PBA, which alleviates ER stress in both cell lines and animal models [60], reduced apoptosis and SA-β-Gal activity and increased cell viability in senescent chondrocytes [34].

These in vitro models provide a controlled and reproducible environment to investigate the cellular and molecular changes associated with senescence, ultimately contributing to our understanding of age-related musculoskeletal diseases and potential therapeutic interventions. However, it is important to note that in vitro models have their limitations, and findings from these models must be further validated using in vivo studies for translations to human health.

Indeed, studying senescence in musculoskeletal tissues and cells using in vitro models has still some limitations, which can impact the translatability and relevance of the findings to the complex in vivo environment. Some of these limitations include the lack of: (1) tissue complexity, which can lead to oversimplification of the biological processes involved in senescence; (2) cellular heterogeneity; (3) mechanical loading; (4) immune system interactions; and (5) standardized protocols.

In addition, a short-term culture may not capture the long-term effects and progression of senescence observed in aging musculoskeletal tissues and may not fully capture the genetic and epigenetic changes that occur during aging and senescence in vivo.

Many senescence studies use immortalized cell lines, which may not fully represent the senescence process in primary cells. Using primary cells more often can provide more physiologically relevant insights into senescence mechanisms.

Developing 3D models for senescence studies can provide more physiologically relevant data, and the combination of genomics, transcriptomics, proteomics, and metabolomics can provide a comprehensive view of senescence.

While in vitro models are valuable, they should be validated in animal models to confirm the relevance of findings in vivo. This step is critical for translating laboratory discoveries into potential clinical applications.

However, in vivo and clinical studies have their advantages and limitations, and they complement each other in aging research. The advantages in the in vivo models concern complexity, the possibility to perform longitudinal studies, and genetics, whereas the limitations regard species differences, ethical concerns, and high costs and time consumption. The advantages of human aging studies are their direct relevance, ethical considerations, and the possibility to perform clinical observations, but the main limitations are the ethical and practical constraints; the fact that long-term human aging studies can be time consuming, expensive, and subject to dropouts; and genetic diversity.

In summary, in vitro models are useful for controlled experiments and initial insights, animal models allow for more complex biological contexts and genetic manipulation, and human studies are essential for understanding human-specific aging processes and interventions. A combination of these approaches, along with advanced computational modeling, can provide a more comprehensive understanding of the aging process and improve the development of strategies to promote healthy aging.

## 5. Conclusions

Studies included in the present systematic review used in vitro models of senescence in musculoskeletal tissues, providing powerful tools to analyze the complex processes of aging in musculoskeletal tissues and to provide valuable insights into age-related changes and pathologies, ultimately contributing to the development of new therapeutic approaches to mitigate the effects of aging on musculoskeletal health.

The cells and tissues that were mostly investigated were those obtained from IVD, followed by cartilage and muscle tissues.

The most employed method to induce cell senescence was the addition of inflammatory cytokines in the culture medium, followed by irradiation and compression. The addition of H_2_O_2_ was relatively limited.

Unfortunately, there are still few studies focused on therapeutic treatments including resveratrol, 4-PBA, and E2; therefore, it would be desirable that there will be more in vitro studies investigating therapeutic treatments in future research.

## Figures and Tables

**Table 1:** Summary of the results of the included studies.

Cells/Tissue	In Vitro Model of Senescence	Evaluations	Main Results	Ref.
NP cells from healthy SD rats (6–8 wks old)	Group 1: cells; Group 2: cells + TNFα (20 ng/mL) and IL1β (20 ng/mL)	Cell proliferation (CCK8 assay); Cell senescence (SA-β-Gal staining); Cell cycle (Flow cytometry); TE activity (ELISA); ROS content (ROS assay); AGG, COLL II, p53, p16, MMP3, MMP13, ADAMTS4 gene expression (RT-PCR); AGG, COLL II proteins (ICC); p16, p53 proteins (WB)	Group 2: ↑ ROS content, SA-β-Gal, G0/G1 phase, p16, p53, MMP3, MMP13 and ADAMTS4 expression; ↓ cell viability, TE activity, S phase, AGG, and COLL II proteins than group 1	Li et al., 2019 [19]
NP cells and discs from healthy SD rats (6–8 wks old)	Group 1: cells; Group 2: cells + TNFα (10 ng/mL); Group 3: discs; Group 4: discs + TNFα (200 ng/mL)	Cell proliferation (CCK8 assay); Cell senescence (SA-β-Gal staining); Cell cycle (Flow cytometry); TE activity, ROS content (ELISA); p16, p53, AGG, COLL II gene expression (RT-PCR); AGG, COLL II, ERα, ERβ proteins (ICC); p16, p53, AGG, COLL II, NF-κB p65, p-NF-κB p65, ERα, ERβ proteins (WB); GAG, HYP content; AGG, COLL II proteins (IHC)	Groups 2, 4: ↓ cell viability, TE activity, AGG and COLL II proteins; ↑ SA-β-Gal, ROS, and p53 and p16 proteins than groups 1, 3	Li et al., 2017 [20]
NP cells from healthy SD rats (6–8 wks old)	Group 1: cells; Group 2: cells + TNFα (10, 20, 40 ng/mL); Group 3: cells + TNFα (10 ng/mL) + recovery in normal culture conditions; Group 4: cells + TNFα (10 ng/mL) without recovery	Cell proliferation (CCK8 assay); Cell viability (LIVE/DEAD); Cell senescence (SA-β-Gal staining); Cell cycle (Flow cytometry); TE activity (ELISA); p53, p16, AGG, COLL II, AKT gene expression (RT-PCR); GAG content (DMMB assay); AGG, COLL II proteins (ICC)	Groups 2, 4: ↓ cell proliferation, TE activity, AGG and COLL II proteins; ↑ p53, p16, and AKT expression than group 1 in a dose-dependent manner. Group 3: similar results than group 4	Li et al., 2017 [21]
NP cells from healthy SD rats (4 wks old); BMSCs from healthy SD rats (4–5 days old)	Group 1: cells + blank calcium alginate gel balls; Group 2: cells treated with TNFα (20 ng/mL) + blank calcium alginate gel balls; Group 3: cells treated with TNFα (20 ng/mL) + calcium alginate gel balls with BMSCs; Group 4: cells treated with TNFα (20 ng/mL) + calcium alginate gel balls with NP cells	Cell viability (CCK8 assay); Cell senescence (SA-β-Gal staining); Cell cycle (Flow cytometry); p16, p21, p53 gene expression (RT-PCR); COLL II, MMP9, ZMPSTE24, RelA proteins (IF)	Groups 2, 4: ↑ SA-β-Gal than groups 1, 3. Groups 2, 4: ↑ SA-β-Gal, MMP9 protein, p16, p21, and p53 expression; ↓ ZMPSTE24 protein than group 3	Li et al., 2019 [22]
Discs of healthy pigs (3–4 mo old)	Group 1: discs; Group 2: discs + intermittent compression (0.1 MP at 1.0 Hz for 2 hrs) + perfusion-culture; Group 3: discs + intermittent compression (1.3 MP at 1.0 Hz for 2 hrs) + perfusion-culture	Cell senescence (SA-β-Gal staining); PG distribution (Alcian Blue staining); AGG, COLL II, TIMP1, TIMP3, ADAMTS4, MMP3, p16, p53 gene expression (RT-PCR); p38, p-p38, p16, p53 proteins (WB); AGG, COLL II, PCNA proteins (IHC); GAG, HYP content (Biochemical measurement)	Group 3: ↑ SA-β-Gal, p38, p-p38, p16, p21, p53, ADAMTS4, and MMP3 proteins; ↓ AGG, COLL II, GAG, HYP, and PCNA proteins, TIMP3 expression than groups 1, 2	Pang et al., 2017 [23]
NP cells from healthy SD rats (7–8 wks old), suspended in collagen solution and seeded into DBM	Group 1: cells; Group 2: cells + 20% dynamic compression (1.0 Hz for 6 hrs/day)	Cell proliferation (CCK8 assay); Cell senescence (SA-β-Gal staining); Cell cycle (Flow cytometry); TE activity, ROS content (ELISA); p16, p53 gene expression (RT-PCR); p16, p53, NF-κB p65, p-NF-κB p65 proteins (WB)	Group 2: ↓ cell proliferation; ↑ SA-β-Gal, p53, p16, and NF-κB proteins, ROS content than group 1	Jiang et al., 2018 [24]
Discs from healthy pigs (12–13 mo old)	Group 1: discs; Group 2: discs + static compression (0.4 MP at 1.0 Hz for 4 hrs/day); Group 3: discs + dynamic compression (0.4 MP at 1.0 Hz for 4 hrs/day)	Cells senescence (SA-β-Gal staining); TE activity (ELISA); AGG, COLL II, p16, p53 gene expression (RT-PCR); COLL II, AGG proteins (IHC); p53, p16 proteins (WB); GAG, HYP content (Biochemical measurement)	Group 2: ↑ SA-β-Gal, p16 and p53 proteins; ↓ TE activity, AGG, COLL II, GAG, and HYP proteins than groups 1, 3. Group 3: ↓ SA-β-Gal, p16 and p53 proteins; ↑ TE activity, AGG, COLL II, GAG, and HYP proteins than groups 1, 2	Shi et al., 2018 [25]
AF cells from healthy SD rats (3 mo old)	Group 1: cells; Group 2: cells + 5% elongation (1 Hz for 8 hrs/day); Group 3: cells + 20% elongation (1 Hz for 8 hrs/day)	Cell proliferation (CCK8 assay); Cell cycle (Flow cytometry); TE activity (ELISA); AGG, COLL II, BCL1, LC3, p53, p16 gene expression (RT-PCR); p16, p53, AGG, COLL II, BCL1, LC3 proteins (WB)	Group 1: similar results than group 2; Group 3: ↓ cell proliferation, TE activity, S phase, AGG, COLL II, BCL1, and LC3 proteins; ↑ G0/G1 phase, p16, and p53 proteins than groups 1, 2	Zhao et al., 2019 [26]
NP cells from healthy SD rats (8 wks old)	Group 1: cells; Group 2: cells + H_2_O_2_ (0.05–0.15 mM)	Cell morphology (H/E, toluidine blue staining); Cell proliferation (CCK8 assay); Cell senescence (SA-β-Gal staining); Nuclear morphology (Hoechst staining); ROS level; Cell cycle, apoptosis (Flow cytometry); TNFα, IL1β, IL6, IL8 gene expression (RT-PCR); p53, p21, p16 proteins (WB)	Group 2: ↑ SA-β-Gal, p53, p21, and p16 proteins, TNFα, IL1β, IL6, and IL8 expression; ↓cell viability and proliferation than group 1	He et al., 2019 [27]
NP cells from healthy subjects (nr)	Group 1: cells; Group 2: cells + H_2_O_2_ (0–2 mM)	ROS kinetics (DCFH-DA assay); Cell viability (MTT assay); Cell proliferation (DNA synthesis assay); Cell cycle (Flow cytometry); Cell senescence (SA-β-gal staining); Telomere length; p16, p21 Catalase, SOD, COX2, ADAMTS4, ADAMTS5, MMP1, MMP2, MMP9, MMP13, TIMP1, TIMP2, TIMP3, ACAN, Biglycan, Decorin, Versican gene expression (RT-PCR); PARP, p-p38, p38, p-SAPK/JNK, SAPK/JNK, p-AKT, AKT, p-Chk2, p-p53, p-ERK1/2, panERK, p21, p-ATM, p53, p16, pRb proteins (WB); p-H2A.X (IF)	Group 2: ↑ ROS; ↓ cell proliferation than group 1. Group 2 at 0.5 mM: ↑ p-38, H2A.X, and p53 proteins, SA-β-gal, ROS, and ECM-degrading enzymes; ↓ ECM structural components	Dimozi et al., 2015 [28]
NP and AF cells of healthy Wistar rats (2 mo old); NP and AF cells from healthy Wistar rats (2 and 22 mo old); NP and AF cells from pz with disc herniation (nr); NP and AF cells from healthy subjects (nr)	Group 1: cells from pz; Group 2: cells from pz + γ-ray (^60^Co gamma, 2 Gy/min); Group 3: cells from young rats + γ-ray (^60^Co gamma, 2 Gy/min); Group 4: cells from old rats + γ-ray (^60^Co gamma, 2 Gy/min); Group 5: cells from healthy individuals + γ-ray (^60^Co gamma, 2 Gy/min)	Cell proliferation (BrdU incorporation assay); Cell senescence (SA-β-gal staining); GL13 protein (ICC); p16, GL13 proteins (IHC)	Group 2: ↓ cell proliferation; ↑ SA-β-gal, GL13 proteins than group 1. Group 4: ↑ SA-β-gal, GL13 protein than group 3. Group 2: ↑ p16, GL13 proteins than group 5	Veroutis et al., 2021 [29]
NP cells from healthy bovine (nr)	Group 1: cells; Group 2: cells + 10% FBS, 4.5 mg/mL glucose, 300 mOsm/kg H_2_O, 20% O_2_ + γ-ray (^60^Co gamma, 2.5 Gy/min); Group 3: cells + serum-free, 0.9 mg/mL glucose, 400 mOsm/kg H_2_O, 2% O_2_ + γ-ray (^60^Co gamma, 2.5 Gy/min)	Cell proliferation (BrdU incorporation); Cell senescence (SA-β-Gal staining); p16, p21, ICAM1, MMP1, MMP2, MMP3, ADAMTS4, ADAMTS5, AGG, COLL II gene expression (RT-PCR)	Group 2: ↓ cell proliferation, ↑ SA-β-Gal than group 1. Groups 2, 3: ↑ p16, p21, ICAM1, MMP1, MMP2, MMP3, AGG, and MMP1 expressions; ↓ COLL II expression than group 1	Kouroumalis et al., 2019 [30]
Purchased human Chons	Group 1: cells + monoenergetic C-ions (75–95 MeV/mm); Group 2: cells + X-ray (225 kV, 13 mA); Group 3: cells in collagen scaffold + monoenergetic C-ions (75–95 MeV/mm); Group 4: cells in collagen scaffold + X-ray (225 kV, 13 mA)	Cell proliferation (Flow cytometry); Cell toxicity (Bioluminescent cytotoxicity assay); Cell senescence (SA-β-gal staining); Cell apoptosis (Flow cytometry); COX2, p21, Caspase-3 proteins (WB)	Group 1: ↓ cell viability than group 2. Groups 1, 3: ↑ SA-β-gal than groups 2, 4. Groups 3, 4: ↑ p21; ↓ COX2 than groups 1, 2	Hamdi et al., 2015 [31]
Chons from healthy horses (4–7 yrs old); Chons from cadavers (38–73 yrs old)	Group 1: cells; Group 2: cells + γ-ray (10 Gy); Group 3: cells + TGFβ1 and bFGF; Group 4: cells + γ-ray (10 Gy) + TGFβ1 and bFGF	Cell senescence (SA-β-gal staining); MMP13, IL6, IGFBP3, CCL2 gene expression (RT-PCR); p16, γH2AX proteins (IF)	Group 2: ↑ SA-β-gal, p16, γH2AX than group 1; Group 4: ↑ SA-β-gal, γH2AX, and p16 proteins, IGFBP3 and CCL2 gene expressions than groups 2, 3	Copp et al., 2021 [32]
BMSCs from healthy SD rats (3 wks old); Chons from healthy SD rats (3 wks old)	Group 1: BMSCs; Group 2: Chons; Group 3: BMSCs + Chons; Group 4: Chons + γ-ray (1.080 Gy/min for 10 min); Group 5: BMCs + Chons + γ-ray (1.080 Gy/min for 10 min)	Cell proliferation (EdU incorporation assay); Cell senescence (SA-β-Gal staining); Bax, Bcl-2, CDKN2A, CDKN1A, IL6, MMP1, MMP13, Sox9, IL1β, Coll II, Nanog, Oct-4 gene expression (RT-PCR); Caspase-3 protein (IHC); COLL II, AGG proteins (WB)	Group 4: ↑ SA-β-Gal, IL6 and MMP13 expression, Caspase-3 protein; ↓ proliferation, Bax/Bcl2, IL1β and COLL II expression than group 2. Group 5: ↓ proliferation, COLL II gene expression and protein; ↑ Caspase-3 protein, Bax/Bcl2, Oct4 and Nanog expression, SA-β-Gal, AGG protein than groups 1, 3	Cao et al., 2019 [33]
Chons from healthy subjects (28–59 yrs old)	Group 1: cells; Group 2: cells cultured for 24 hrs + IL1β (10 ng/mL)	Cell proliferation (CCK8 assay); Cell apoptosis (Flow cytometry); Cell senescence (SA-β-gal staining); COLL II, ADAMTS5, MMP13 gene expression (RT-PCR); GRP78 protein (WB)	Group 2: ↓ COLL II expression; ↑ ADAMTS5 and MMP13 gene expressions, GRP78 protein than group 1	Liu et al., 2017 [34]
Chons from NZW rabbits (1 mo old) and rats (2 wks old)	Group 1: rabbit cells + serum-starved overnight conditions + DKK1 (100 ng/mL) + IL1β (10 ng/mL); Group 2: rabbit cells + serum-starved overnight conditions + LiCl (20 mM) + IL1β (10 ng/mL); Group 3: rabbit cells; Group 4: rat cells + serum-starved overnight + Wnt-1 (10 ng/mL); Group 5: rat cells; Group 6: rat cells + serum-starved overnight + Wnt-1 (10 ng/mL) + IL1β (10 ng/mL); Group 7: rat cells + serum-starved overnight + Wnt-1 (10 ng/mL) + PBS	Cell senescence (SA-β-gal staining); MMP13, p53, 18s rRNA, MMP3 gene expression (RT-PCR); β-catenin, MMP13, MMP3, p16, p53, acetylated p53, SIRT-1 proteins (WB)	Group 2: ↑ β-catenin, MMP13, and acetylated p53, SIRT-1 proteins than group 1. Group 4: ↑ cell senescence, p53, and p16 proteins than group 5. Group 6: ↑ MMP3 and MMP13 proteins than group 7	Li et al., 2020 [35]
Chons from cartilage of preserved (P-C) and severely damaged (D-C) areas of OA pz (nr)	Group 1: Chons micromasses from P-C; Group 2: Chons micromasses from D-C	Cell proliferation (Flow cytometry); Cell senescence (SA-β-gal staining); Telomere length (TAT assay); p16INK4a, p21, p53, COLL II, SOX9, AGG, Ats4, Ats5, MMP12, MMP13, COLL I, COLL III, VCAN, IL6, IL8, p16, SA-β-gal gene expression (RT-PCR); p16INK4a, MMP13 proteins (IHC); COLL II, p16INK4a proteins (IF); SOX9, MMP13, p21, p53, p16 proteins (WB); GAG content (Safranin O/Fast green staining)	Group 2: ↓ cartilage matrix deposition, telomere length, COLL II, SOX9, and AGG expression; ↑ MMP13, p16, p21, and p53 proteins and expressions than group 1	Wang et al., 2022 [36]
Purchased C2C12 (10th–12th in vitro passages)	Group 1: myotubes; Group 2: myotubes + 10% of serum of fasted young donors; Group 3: myotubes + 10% of serum of old donors	Desmin protein (ICC); IgG2a monoclonal antipuromycin, p-mTOR, mTOR, p-Akt, Akt, p-p70S6K1, p70S6K1, p-4EBP-1, 4EBP1, p-RPS-6, RPS-6, p-eEF2, eEF2, LC3A/B, Caspase-3, MuRF-1, F-box (WB)	Group 2: ↑ myotube diameter and NFI, Akt, p70S6K1, and eEF2 proteins than groups 1, 3. Group 3: ↓ Myotube diameter than group 1	Allen et al., 2021 [37]
Purchased C2C12 cells	Group 1: cells + 5% plasma from young subjects; Group 2: cells + 5% plasma from old subjects	Cell proliferation (cell counter); Scratch assay; Cell diameter	Group 1: ↓ scratch size; ↑ cell diameter, proliferation than group 2	Kalampouka et al., 2018 [2]
Purchased human myoblasts	Group 1: cells at 1st passage + simulated microgravity (1 × 10^−3^ G; µG); Group 2: cells at 1st passage + normal gravity (1 G); Group 3: cells at 5th passage + normal gravity (1 G)	Cell proliferation assay; Cell senescence (SA-β-Gal activity); Desmin, Myf5 proteins (ICC); Cytoskeleton areas and nuclei analysis; Differentiation into myotubes (IF for MHC)	Groups 2, 3: ↑ MHC and desmin proteins, myotubes diameter, cell proliferation; ↓ Myf5 protein than group 1. Groups 1, 3: ↑ SA-β-Gal than group 2	Takahashi et al., 2021 [38]
BMSCs from OA pz	Group 1: cells at 10th passage; Group 2: cells at 4th passage	Tri-lineage differentiation; CFU assay; Cell proliferation (Flow cytometry, MTT); Cell senescence (SA-β-gal staining); Telomere length (TAT assay); p16, MMP13 proteins (IHC); p16, p21, p53, COLL II, SOX9, AGG, Ats4, Ats5, MMP12, MMP13, COLL I, COLL III, VCAN, IL6, IL8, SA-β-gal gene expression (RT-PCR); COLL II, p16 proteins (IF); SOX9, MMP13, p21, p53, p16INK4a proteins (WB)	Group 1: ↓ proliferation, differentiation potential, telomere length, COLL II, SOX9, and AGG expressions and proteins; ↑ p16, SA-β-gal, MMP13, p21, and p53 expressions and proteins than group 2	Wang et al., 2022 [36]
NPMSCs from pz (37–52 yrs old)	Group 1: cells; Group 2: cells + TNFα (10 ng/mL)	Cell viability (Calcein-AM); Cell senescence (SA-β-Gal staining); Cell proliferation (CCK8 assay); p16, p21, p53, IL6, MMP13, ADAMTS5, brachyury, FOXF1, Pax1, CA12, HIF1α, COLL Iα1, COLL IIα1, SOX9, AGG, LPL, PPAR2, ALP, Runx2 gene expression (RT-PCR); COLL II, CA12 proteins (IF, WB); GAG content (DMMB assay)	Group 2: ↑ senescent cell morphology, SA-β-Gal, p16, p21, p53, IL6, MMP13, and ADAMTS5 expressions than group 1	Li et al., 2018 [39]

Abbreviations: Ref = references; NP = nucleus pulposus; SD = Sprague–Dawley; wks = weeks; TNFα = Tumor necrosis factor α; IL = interleukin; CCK8 = cell counting kit-8 reagents; Sa-β-Gal = Senescence-associated β-galactosidase; TE = telomerase; ELISA = enzyme-linked immunosorbent assay; ROS = reactive oxygen species; AGG = aggrecan; COLL = collagen; MMP = matrix metalloproteinase; ADAMTS = a disintegrin and metalloproteinase with thrombospondin motifs; RT-PCR = real time PCR; ICC = Immunocytochemistry; WB = Western blotting; GAG = glycosaminoglycan; HYP = hydroxyproline; IHC = Immunohistochemistry; ER = estrogen receptor; DMMB = dimethylmethylene blue; BMSCs = bone marrow mesenchymal stem cells; ZMPSTE24 = zinc metallopeptidase STE24; IF = immunofluorescence; mo = months; hrs = hours; PG = proteoglycan; TIMP = tissue inhibitor of metalloproteinase; PCNA = proliferating cell nuclear antigen; NF-κB = nuclear factor kappa-light-chain-enhancer of activated B cells; AF = Annulus Fibrosus; H_2_O_2_ = hydrogen peroxide; BrdU = 5-bromo-2′-deoxyuridine; Chons = chondrocytes; COX-2 = cyclooxygenase-2; yrs = years; bFGF = basic fibroblastic growth factor; TGFβ = transforming growth factor beta; IGFBP3 = insulin-like growth factor binding protein 3; CCL2 = C-C motif chemokine ligand 2/monocyte chemoattractant protein 1; GRP78 = 78-KDa glucose-regulated protein; NZW = New Zealand white; NPMSCs = nucleus pulposus mesenchymal stem cells; DBM = decalcified bone matrix; DCFH-DA = Dichloro-dihydro-fluorescein diacetate; ECM = extracellular matrix; pz = patients; ICAM1 = intercellular adhesion molecule 1; Oct-4 = octamer-binding transcription factor 4; CDKN = cyclin dependent kinase inhibitor; LiCl = Lithium chloride; PBS = Phosphate buffered saline; SIRT-1 = Sirtuin-1; DKK1 = Dickkopf-1; OA = osteoarthritis; TAT = telomere analysis technology; EEF2 = Eukaryotic Translation Elongation Factor 2; NFI = Nuclear Factor I; MuRF1 = Muscle RING-finger protein-1; RPS6 = Ribosomal Protein S6; EIF4EBP1 = Eukaryotic Translation Initiation Factor 4E Binding Protein 1; µG = microgravity; G = gravity; MYF5 = Myogenic Factor 5; MHC = Myosin heavy chain; CFU = colony-forming unit; ALP = alkaline phosphatase; RUNX2 = Runt-related transcription factor 2; PPAR = peroxisome proliferator-activated receptors; LPL = Lipoprotein lipase; HIF = Hypoxia-inducible factor; H/E= Hematoxylin/Eosin; CA12 = Carbonic anhydrase 12; nr = not reported; ↑ = increase; ↓ = decrease.

**Table 2:** Summary of anti-senescence treatments of the included studies.

Group (CTR)	Treatment	Main Results	Ref.
NP cells + TNFα (20 ng/mL) and IL1β (20 ng/mL)	NP cells + TNFα (20 ng/mL) and IL1β (20 ng/mL) + resveratrol (100 µM)	Resveratrol: ↓ ROS, SA-β-Gal, G0/G1 phase, p16, p53, MMP3, MMP13, and ADAMTS4 proteins; ↑ cell proliferation, S phase, TE activity, AGG, and COLL II proteins than CTR	Li et al., 2019 [19]
NP cells + 20% dynamic compression (1.0 Hz for 6 hrs/day)	NP cells + 20% dynamic compression (1.0 Hz for 6 hrs/day) + resveratrol (30 μM or 60 µM)	Resveratrol: ↑ cell proliferation; ↓ SA-β-Gal, p53, p16, and NF-κB proteins, ROS content than CTR	Jiang et al., 2018 [24]
NP cells or discs + TNFα (10 ng/mL)	NP cells or discs + TNFα (10 ng/mL) + E2 (10^−7^ M); NP cells + TNFα (10 ng/mL) + E2 (10^−7^ M) + ICI 182,780 (10 μM)	E2: ↑ cell proliferation, TE activity, AGG and COL II proteins; ↓ SA-β-Gal, ROS, p53, and p16 proteins than CTR and E2 + ICI182780	Li et al., 2017 [20]
NPMSCs + TNFα (10 ng/mL)	NPMSCs + TNFα (10 ng/mL) + rabbit NC-rich NP explant	NC-rich NP explant: ↑ cell proliferation, CA12, FOXF1, Pax-1, AGG, SOX-9, and COLL II proteins; ↓ COLL I protein than CTR	Li et al., 2018 [39]
Chons + IL1β (10 ng/mL)	Chons + IL1β (10 ng/mL) + 4-PBA (100 µM)	4-PBA: ↓ GRP78 protein, SA-β-gal, apoptosis; ↑ cell viability than CTR	Liu et al., 2017 [34]

Abbreviations: Ref = references; NP = nucleus pulposus; CTR = control; TNFα = Tumor necrosis factor α; IL = interleukin; ROS = reactive oxygen species; Sa-β-Gal = Senescence-associated β-galactosidase; MMP = matrix metalloproteinase; ADAMTS = a disintegrin and metalloproteinase with thrombospondin motifs; COLL = collagen; AGG = aggrecan; TE = telomerase; hrs = hours; NF-κB = nuclear factor kappa-light-chain-enhancer of activated B cells; Chons = chondrocytes; NPMSCs = nucleus pulposus mesenchymal stem cells; GRP78 = 78-KDa glucose-regulated protein; E2 = 17-beta-estradiol; CA12 = Carbonic anhydrase 12; NC = Notochordal cell; 4-PBA = 4-phenylbutyric acid; ↑ = increase; ↓ = decrease.

## Data Availability

Not applicable.
